# Development of a high-copy plasmid for enhanced production of recombinant proteins in *Leuconostoc citreum*

**DOI:** 10.1186/s12934-015-0400-8

**Published:** 2016-01-15

**Authors:** Yeon Jeong Son, Ae Jin Ryu, Ling Li, Nam Soo Han, Ki Jun Jeong

**Affiliations:** Department of Chemical and Biomolecular Engineering, BK21 Plus PROGRAM, KAIST, 291 Daehak-ro, Yuseong-gu, Daejeon, 34141 Republic of Korea; Division of Animal, Horticultural and Food Sciences, Brain Korea 21 Center for Bio-Resource Development, Chungbuk National University, Cheongju, 28644 Republic of Korea; Institute for the BioCentury, KAIST, 291 Daehak-ro, Yuseong-gu, Daejeon, 34141 Republic of Korea

**Keywords:** Lactic acid bacteria, *Leuconostoc citreum*, High copy plasmid, Plasmid engineering, FACS

## Abstract

**Background:**

*Leuconostoc* is a hetero-fermentative lactic acid bacteria, and its importance is widely recognized in the dairy industry. However, due to limited genetic tools including plasmids for *Leuconostoc*, there has not been much extensive research on the genetics and engineering of *Leuconostoc* yet. Thus, there is a big demand for high-copy-number plasmids for useful gene manipulation and overproduction of recombinant proteins in *Leuconostoc*.

**Results:**

Using an existing low-copy plasmid, the copy number of plasmid was increased by random mutagenesis followed by FACS-based high-throughput screening. First, a random library of plasmids was constructed by randomizing the region responsible for replication in *Leuconostoc citreum;* additionally, a superfolder green fluorescent protein (sfGFP) was used as a reporter protein. With a high-speed FACS sorter, highly fluorescent cells were enriched, and after two rounds of sorting, single clone exhibiting the highest level of sfGFP was isolated. The copy number of the isolated plasmid (pCB4270) was determined by quantitative PCR (qPCR). It was found that the isolated plasmid has approximately a 30-fold higher copy number (approx. 70 copies per cell) than that of the original plasmid. From the sequence analysis, a single mutation (C→T) at position 4690 was found, and we confirmed that this single mutation was responsible for the increased plasmid copy number. The effectiveness of the isolated high-copy-number plasmid for the overproduction of recombinant proteins was successfully demonstrated with two protein models Glutathione-S-transferase (GST) and α–amylase.

**Conclusions:**

The high-copy number plasmid was successfully isolated by FACS-based high-throughput screening of a plasmid library in *L. citreum*. The isolated plasmid could be a useful genetic tool for high-level gene expression in *Leuconostoc*, and for extending the applications of this useful bacteria to various areas in the dairy and pharmaceutical industries.

## Background

Lactic acid bacteria (LAB) are a group of gram-positive, low G + C content food grade bacteria that include the species of *Lactobacillus, Lactococcus, Pediococcus, Streptococcus* and *Leuconostoc* [1]. Since ancient times, LAB have been used for the fermentation of various dairy foods like cheese and yogurts. Besides their traditional uses, LAB are also regarded as attractive hosts for recombinant protein production due to their generally recognized as safe (GRAS) status [2, 3]. *Leuconostoc* is a hetero-fermentative LAB that also has an important role in the fermentation of milk, vegetables, meat and other dairy products [4]. *Leuconostoc* produces flavor-enhancing aromatic compounds in food products [5] and synthesizes dextrans which are used as food additives and industrial products like Sephadex [6]. In addition, *Leuconostoc* has a health-promoting effects including the production of bioactive peptides [7] and vitamins [8]. Compared to other well-used cell factories inculding *Escherichia coli*, *Bacillus subtilis* as well as *Pichia pastoris* and *Saccharomyces cerevisiae*, etc., *L. citreum* is also regarded as an attractive host for the production of recombinant proteins (particularly pharmaceutical proteins or vaccines) due to several distinct advantages; GRAS strain, ability of protein secretin, relatively rapid growth than yeast hosts [3, 9]. Most of all, *L. citreum* is also GRAS status and a food-grade bacteria, so it can be a very attractive host for the delivery of pharmaceutical proteins into human or animals with a higher impact in Pharma and Biotech induestries.

Even though *Leuconostoc* has significant importance in bioindustry, there has not been much extensive research on the genetics and engineering of *Leuconostoc* yet because of a lack of gene expression/manipulation tools. Thus, preliminary, it is necessary to develop key tools such as plasmids, efficient transformation methods, and gene expression systems to make *Leuconostoc* a more promising host for use in a wide variety of applications. In particular, expression plasmids are one of the most fundamental systems for genetic manipulations and protein production, especially to carry foreign DNA and to produce recombinant proteins in a host. In the last decades, numerous studies have focused on the development of cloning plasmids for use in LAB [10], and a few plasmids have been successfully developed for the *Leuconostoc* genus [11–13]. While most of them are useful for gene cloning and transformation, they are not suitable for high-level production of recombinant proteins because of their low replication copy number. Therefore, a useful plasmid with an increased copy number first needs to be developed which would enable further extensive genetic studies in *Leuconostoc* and its engineering.

In this work, we developed a high-copy plasmid suitable for enhanced production of recombinant proteins in *L. citreum* CB2567 (KACC 91348P). For this purpose, we first constructed a random library of plasmids by randomization of the replication region with the superfolder green fluorescent protein (sfGFP) as a reporter protein. The random library was screened with the FACS-based high-throughput screening tool, and the most fluorescent cell was successfully isolated. The isolated plasmid with a high-copy number was characterized, and its usefulness in the overproduction of recombinant proteins in *L. citreum* was evaluated by examining the production of two model proteins, GST and α-amylase.

## Results

### Construction of the plasmid library

**Fig. 1:**
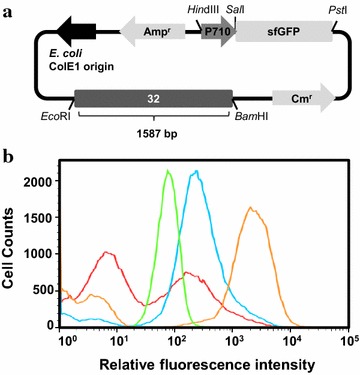
Library construction and FACS screening. **a** Schematic diagram of plasmid pCB4170-sfGFP. Library was constructed by the randomization of the 32-region (1587 bp) related to the plasmid replication. **b** The histogram of FACS screening. *L. citreum* harboring the original pCB4170-sfGFP (*green curve*) was used as a control. The original library, 1st round sorted library and 2nd round sorted library are indicated in histogram by *red*, *blue* and *orange*, respectively

For the library construction, the pCB4170-sfGFP, which is an *E. coli*-*L. citreum* shuttle vector with sfGFP expression under the constitutive P710 promoter, was used as the template DNA. The pCB4170 plasmid is a derivative of pLeuCM42 that combines the pUC plasmid origin in *E. coil* and the origin of *L. citreum* cryptic plasmid pCB42 [13]. In pCB4170-sfGFP, the 32-region (1587 bp) between the 3.9 Kb to 5.5 Kb position, which originates from pCB42 from *L. citreum* CB2567 (Fig. 1a), is responsible for the replication of the plasmid in *L. citreum* [12, 14], and that 32-region was randomized by error-prone PCR with a 0.2 % error rate (Fig. 1a). The random library was first constructed in *E. coli*, and after purification, the plasmids were transformed to *L. citreum* CB2567. Finally a library of 1 × 10^5^ cells was constructed in *L. citreum.* From the library, 30 clones were randomly picked, and the sequences of the randomized region were determined by DNA sequencing experiments, and it was confirmed that each clone contained 1–4 mutations (0.1–0.2 % error rate) (data not shown).

### Library screening by FACS

**Fig. 2:**
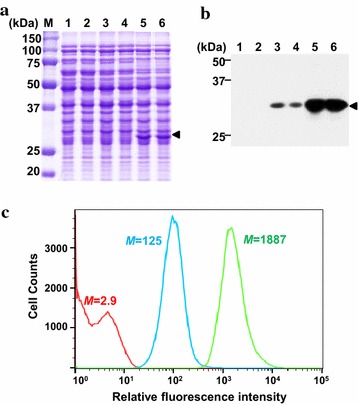
Analysis of sfGFP expression in *L. citreum* by **a** SDS-PAGE and **b** western blotting. Protein samples (total and soluble protein fractions) were taken at 8 h cultivation. Lane M, molecular weight markers (kDa); *lanes 1* and *2*, pCB4170; *lanes 3* and *4*, pCB4170-sfGFP; *lanes 5* and 6, pCB4270-sfGFP; *lanes 1*, *3* and *5*, total protein fractions; *lanes 2*, *4* and *6*, soluble protein fraction. The* arrowheads* indicate the band of sfGFP. **c** The histogram of FACS analysis. pCB4170, pCB4170-sfGFP and pCB4270-sfGFP are represented in histogram by* red*,* blue* and* green*, respectively. M values indicate the mean fluorescent intensity

For the isolation of a high-copy plasmid, the random library was screened with a high-speed FACS sorter. In the first round, the population with high fluorescent signals (top 3 % of the total cells) were selectively sorted (Fig. 1b), and the collected cells were spread on MRS agar plates to reduce growth retardation of positive clones. In the second round of sorting, the top 1 % (ca. 50,000 count cells) of the total cells were sorted, and then, the sorted cells were spread onto MRS agar plates. After incubation, 200 individual clones were randomly selected and cultivated in 96 deep well plates for individual analysis. Among the 200 clones, 20 clones were selected, which exhibited higher fluorescent signals than that of the other clones, and the fluorescent signal of each clone was further analyzed by flow cytometer (data not shown). Finally, one clone with the highest signal was selected, and the plasmid in the isolated clone was named pCB4270-sfGFP. In the isolated clone harboring pCB4270-sfGFP, the expression level of sfGFP (27 kDa) was analyzed by SDS-PAGE and western blotting, and compared with those of *L. citreum* harboring the original copy-number plasmids (pCB4170 or pCB4170-sfGFP). In the SDS-PAGE analysis, the band of sfGFP could be highly detected in the *L. citreum* harboring a pCB4270-sfGFP (Fig. 2a). The expression level of sfGFP with cells carrying a pCB4270-sfGFP showed 17-fold increased than that of cells harboring a pCB4170-sfGFP by densitometric analysis. This significantly improved result was also clearly confirmed by western blotting (Fig. 2b). The cell harboring the original pCB4170-sfGFP had much less production of sfGFP (Fig. 2). The fluorescent intensity in each cell was also analyzed by flow cytometer, and the results agreed well with the results from SDS-PAGE and western blotting analysis (Fig. 2c). The cells harboring a pCB4270-sfGFP had a 15-fold higher fluorescent intensity than that of the cells harboring a pCB4170-sfGFP.

### Characterization of the isolated plasmid

**Fig. 3:**
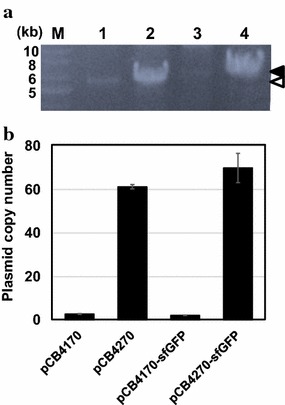
Analysis of plasmid copy number in *L. citreum*. **a** Agarose gel electrophoresis of plasmids. After preparation of plasmids from *L. citreum*, all plasmids were digested by *Bam*HI for linearization of plasmids. *Lane* M, DNA size marker (kb), *lane 1*, pCB4170; *lane 2*, pCB4270; *lane 3*, pCB4170-sfGFP; *lane 4* pCB4270-sfGFP. The* open* and *closed arrowheads* indicate the pCB4270 and pCB4270-sfGFP, respectively. **b** Relative copy number value of pCB4170, pCB4270, pCB4170-sfGFP and pCB4270-sfGFP determined by quantitative PCR method

To determine whether the high-level production of sfGFP in the isolated clone is related to the plasmid copy number, the copy number of pCB4270-sfGFP was analyzed using two methods: (1) plasmid preparation followed by agarose gel electrophoresis and (2) quantitative PCR (qPCR). In these analyses, the pCB4270 plasmid was also examined which was constructed by deleting the sfGFP gene in the pCB4270-sfGFP. After flask cultivation, the plasmids were prepared from the same amount of cells, and the same volume of plasmid samples was loaded into a 0.7 % agarose gel. As shown in Fig. 3a, the plasmids pCB4270 and pCB4270-sfGFP are clearly seen but not the plasmids pCB4170 and pCB4170-sfGFP. This result meant that the isolated plasmid pCB4270-sfGFP and pCB4270 have a much higher copy number than that of the original plasmids pCB4170 and pCB4170-sfGFP. Next, the relative plasmid copy number (PCN) was also measured using the quantitative PCR (qPCR) method. The standard curve of each gene had high linearity (R^2^ = 0.99), and the calculated amplification efficiencies of the phosphoketolase (*pho*) and chloramphenicol (Cm) genes, as references, were 2.05 and 2.04, respectively. As a result, the relative copy number of pCB4170, pCB4170-sfGFP, pCB4270, pCB4270-sfGFP was determined as 2.83 ± 0.18, 2.21 ± 0.07, 61.23 ± 1.01, and 69.89 ± 6.74, respectively (Fig. 3b). Based on this analysis, we concluded that the isolated plasmid (pCB4270) has approximately a 30-fold higher copy number than that of the original pCB4170.

### Sequence analysis of pCB4270

**Fig. 4:**
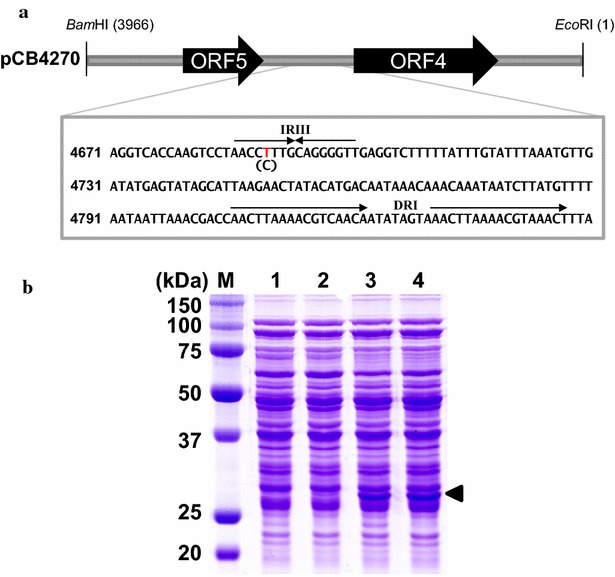
Determination of mutation point by sequence analysis. **a** The 1587 bp region containing two ORFs was randomized and point mutation was found at 4690 bp position which is located in inverted repeat between two ORFs. **b** Analysis of sfGFP expression by SDS-PAGE.* Lane M*, protein size marker (kDa); *lane 1* pCB4170; *lane 2* pCB4170-sfGFP, *lane 3* pCB4270-sfGFP; *lane 4* pCB4170-4690T-sfGFP. The *arrowhead* indicates the sfGFP

The nucleotide sequences of the randomized region in the pCB4270 plasmid was determined with one mutation (C→T) at the 4690th position compared with that of the original pCB4170 (Fig. 4a). The mutation point was located in the third inverted repeat (IRIII) between two ORFs (ORF4 and ORF5). To confirm whether this single mutation contributed to the increase in the plasmid copy number, the same mutation was introduced into the original pCB4170 yielding pCB4170-C4690T. The pCB4170-C4690T was transformed into *L. citreum,* and the copy number was compared with pCB4170 and pCB4270. As expected, the plasmid pCB4170-C4690T also has the almost same copy number as pCB4270-sfGFP. Additionally, sfGFP expression in pCB4170-C4690T was analyzed, and the same increased production yield as that of pCB4270-sfGFP was observed by SDS-PAGE analysis (Fig. 4b). Taken all together, we concluded that the single mutation (C→T) at the 4690th position was responsible for the increase in the copy number in the isolated plasmid.

### Segregational stability and effect on cell growth

**Fig. 5:**
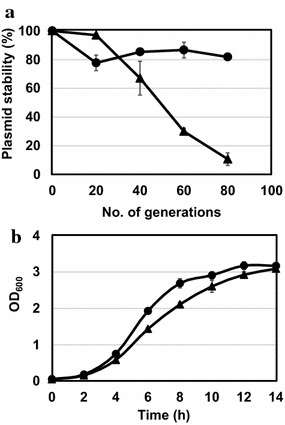
Analysis of segregational stability and cell growth. **a** Analysis of segregational stability of plasmid pCB4170 and pCB4270 during the cultivation of *L. citreum* CB2567 in antibiotic-free media. **b** Growth curve of *L. citreum* CB2567 harboring either pCB4170 or pCB4270 in the medium containing antibiotic (Cm). *Symbols* *Circle*, pCB4170; *Triangle* pCB4270

The segregational stabilities of pCB4270 and pCB4170 were compared by culturing cells under non-selective growth conditions (no antibiotic). The maintenance of each plasmid in *L. citreum* was calculated by the number of colonies grown on MRS plates with chloramphenicol divided by the number of colonies grown on MRS plates without chloramphenicol until 80 generations (10 generations correspond to a 12 h cultivation). The original pCB4170 plasmid exhibited high segregational stability with 80 % of its plasmid present until 80 generations (Fig. 5a). In contrast, the high-copy-number pCB4270 exhibited a relatively lower stability. Up until 40 generations, pCB4270 was maintained with high stability (over 60 %), but this stability rapidly decreased to below 10 % after 80 generations (Fig. 5a).

To determine the effect of the high-copy-number plasmid on cell viability, cells harboring either the high-copy pCB4270 or the original pCB4170 were cultivated in media containing antibiotic (Cm). For a 14-h cultivation, both cells showed very similar growth patterns with similar growth rates: the specific growth rate of cells harboring pCB4170 and pCB4270 were 0.18 h^−1^ and pCB4270 was 0.17 h^−1^, respectively. No deleterious effects on cell growth from the high-copy plasmid were also observed in the cells harboring pCB4270 (Fig. 5b).

### Overproduction of recombinant proteins with high-copy-number plasmid

**Fig. 6:**
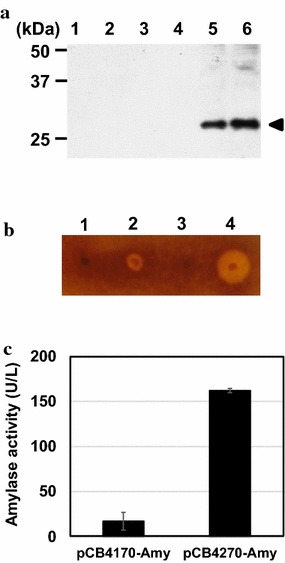
Production of GST and α-amylase in *L. citreum* CB2567 harboring pCB4270-GST or pCB4270-Amy. **a** Western blot analysis of total and soluble fractions of *L. citreum*. *Lanes 1* and *2*, pCB4170; *lanes 3* and *4*, pCB4170-GST; *lanes 5* and *6*, pCB4270-GST; *lanes 1*, *3* and *5*, total protein fractions; *lanes 2*, *4* and *6*, soluble protein fraction. The *arrowhead* indicates GST protein **b** Detection of α-amylase activity on 0.5 % starch MRS-chloramphenicol plate. After incubation, the halos on the plate were visualized by staining with iodine solution. Spots 1 to 4, *L. citreum* CB2567 harboring pCB4170, pCB4170-Amy, pCB4270 and pCB4270-Amy, respectively. **c** Quantitative assay of α-amylase activity using EnzChek^®^ Ultra Amylase Assay Kit. All experiments were done in triplicate

To verify the usefulness of the high-copy-number plasmid for the overproduction of recombinant proteins in *L. citreum*, the production of two different proteins glutathione-S-transferase (GST) and α-amylase was examined. To show the versatility of the high-copy plasmid, we first adopt GST as a small size cytoplasmic protein and also chose α-amylase as a large size secretory protein. First, GST gene expression was constructed in the original pCB4170 and the high-copy pCB4270, and after flask cultivation, the production levels in *L. citreum* were compared by western blotting. As shown in Fig. 6a, the use of the high-copy pCB4270 resulted in a much enhanced production of GST (26 kDa) in the cytoplasm of *L. citreum*, and the production of GST in the original pCB4170 was not detected. Next, the secretory production of α-amylase (105 kDa), which can degrade starch to glucose, was also examined. For the secretory production, α-amylase own signal peptide was used, and the production systems were also constructed in the original pCB4170 and the high-copy plasmid pCB4270. Cells harboring each plasmid were spotted onto starch-containing agar plates, and after incubation for 24 h, the hydrolysis of starch was compared. All the cells grew well on the agar plates, and clear halo zones were visualized by iodine staining. As shown in Fig. 6b, the cells harboring the pCB4270-Amy had much larger halo than those of the controls, and this result indicates that much more α-amylase was expressed and secreted into the extracellular medium when the high-copy plasmid was used. Additionally, the α-amylase enzyme activity was quantitatively measured with an amylase assay kit. Cells harboring the high-copy pCB4270-Amy showed approximately a 9.5-fold higher amylase activity (162 U/L) compared to that (17 U/L) of the cells harboring the original plasmid pCB4170-Amy (Fig. 6c).

## Discussion

For the production of recombinant proteins in bacterial hosts, the use of plasmids is highly recommended, which enables the stable expression of the cloned gene and long-term maintenance in the cell [15]. Generally, a typical plasmid system contains several genetic components including a promoter, RBS, transcription terminator, etc., and high-level production of target proteins can be achieved through various genetic factors including the use of a strong promoter, codon optimization, modification of the UTR/TIR sequences, plasmid copy number, etc. [16, 17]. Plasmids are present as multicopies in cells, and the copy number of a plasmid is a critical factor in gene expression. In *E. coli* hosts, many high-copy-number plasmids including the pUC series (>100 copies) and pBR-ori vectors (20–30 copies) have been developed and successfully used for high-level gene expression in *E. coli* [15]. For the expression of heterologous genes in LAB including the *Leuconostoc* species, several plasmids have been developed, but their copy numbers are generally below 20 copies [11–13, 18]. In general, it is not easy to achieve high-level gene expression using those low-copy plasmids, and the limited availability of plasmids has been one of the big hurdles in using LAB as protein production hosts. In this study, we engineered a high copy plasmid using an existing low-copy plasmid with the FACS-based high-throughput screening (HTS) method. From the random library of plasmids, we isolated one plasmid (pCB4270) and confirmed that the isolated plasmid has a significantly increased copy number (up to 70 copies per cell), which is approximately 30-fold higher than that of the original plasmid (pCB4170). From the DNA sequencing, we determined that the high-copy plasmid has only a single point mutation (C → T at the 4690th position). There are several reports on modifying the plasmid copy numbers in other bacterial hosts including *E. coli* [19–21] and *Legionella pneumophila* [22], and in many cases, only a single mutation was responsible for the increase in plasmid copy number. In *E. coli*, the well-known pUC plasmid, which is a derivative of pBR322, has a single point mutation (G → A) at the Rom/Rop-suppressible point in RNA II and that single mutation resulted in an incredible increase in the plasmid copy number (>100 copies per cell) [23]. Another example in *E. coli* is the single mutation in the RNA II promoter of the ColE1 origin of replication [21]. It was suggested that the transcription of the RNA II is increased by a single mutation which leads to an increased copy number [21]. In our study, we introduced random mutations into the region (total 1587 bp) containing two ORFs (ORF4 and ORF5) which is involved in the initial replication of the plasmid, and a single mutation (C → T) was found in the third inverted repeat (IRIII) point between the two ORFs (Fig. 4a). It is known that direct repeats (DRs) and IRs play a key role in the regulation of the theta-type plasmid replication providing a binding region for the Rep protein and chromosomal encoded protein like DnaA [18, 24, 25]. In a previous work [12], the IRIII was determined to be involved in the replication of plasmids because plasmids without an IRIII region cannot replicate in *Leuconostoc*. Based on these previous reports, we predict that the single mutation in IRIII region might be critical to the binding of the replication initiating protein, and the C → T mutation resulted in an increased copy number of the plasmid. To figure out this prediction, the replication initiating proteins such as Rep and DnaA proteins need to be purified, and the biochemical studies with those proteins can provide more useful information, which will be our next work.

Plasmid segregational stability is another important property of a vector for the stable expression of a heterologous gene during long-term cultivation because the generation of plasmid-free cells can lead to a significant loss in productivity [26, 27]. When we checked the segregational stability of the high-copy-number plasmid, unfortunately, it had a relatively lower stability after 40 generations, while the original plasmid (pCB4170) had a higher stability (above 80 %) until 80 generations (Fig. 5a). There are few reports on the relationship between copy number and stability, and thus, we do not know the mechanism behind the deleterious effect of the single mutation on plasmid stability. Although the stability of the plasmid was poor during cultivation in antibiotic-free media, the cells harboring the high-copy plasmid exhibited a similar growth pattern compared with the cells harboring the original low-copy plasmid during the cultivation in the media containing antibiotic (Fig. 5b). Additionally, we confirmed that a similar level of plasmid was maintained during the entire cultivation with the antibiotic (data not shown). These results indicate that the increase in the plasmid copy number was not deleterious to cell viability, and the use of the high-copy plasmid can ensure the overproduction of recombinant proteins during cultivation in antibiotic-containing media which we showed with three recombinant proteins (sfGFP, GST and α-amylase). The segregational stability can also be further improved by optimizing the culture conditions [26] and we will further engineer the high-copy-plasmid for higher stability.

FACS-based screening is emerging as a powerful tool for screening of mutant libraries due to its high sensitivity and ultrahigh-throughput efficiency [28, 29]. This has been applied to a variety of targets including antibody screening, enzyme engineering, whole cell and receptor binding assays and promoter screening [29, 30]. In this study, we successfully showed the usefulness of FACS-based high-throughput screening for the engineering of plasmid copy number. The *L. citreum* random library was screened, and a clone harboring a high-copy-number plasmid (pCB4270-sfGFP) was successfully isolated just in two rounds of screening. To the best of our knowledge, this is the first report about the application of FACS-based screening to the engineering of plasmid copy number and also the first successful example of FACS-based screening using LAB. As we discussed earlier, the promoter strength has also a significant effect on the gene expression. Previously, our group succeeded in the isolation of new synthetic promoters in *Corynebacterium glutamicum*, and with those synthetic promoters, much enhanced gene expression could be achieved in *Corynebacterium glutamicum* [29]. As a following experiment, now we have a similar plan to develop new promoters with enhanced promoter strengths in *Leuconostoc* sp. If we succeed in this engineering, we believe that much potential expression system with high copy number and strong promoter can be developed in *Leuconostoc* sp.

## Conclusions

In summary, we successfully engineered a plasmid copy number by random mutagenesis followed by FACS-based high-throughput screening in *L. citreum*. The isolated plasmid has a single mutation which resulted in a much increased copy number (up to 70 copies per cell) approximately 30-fold higher compared to the original one. The general availability of the high-copy plasmid for the overproduction of recombinant proteins was also verified with three protein models (sfGFP, GST, and α-amylase). The results of this study suggest that the isolated high-copy-number plasmid can be used as a powerful genetic tool for high-level gene expression in *Leuconostoc* sp. However, the use of high-copy plasmid does not always ensure the high-level production of recombinant proteins (particularly big and complex proteins), because the gene expression can be also affected by several factors (promoter strength, codon usage, 5′UTR/TIR sequences, transcription terminators, protein folding etc.) as discussed earlier. In the future, those factors should be considered together for the enhanced production of more recombinant proteins. The development of those genetic tools also extend the application of this useful bacteria to various areas in the dairy and pharmaceutical industries. In addition, as successfully shown here, the strategy of FACS-based screening can be a useful tool in the engineering of *Leuconostoc* as well as other LAB.

## Methods

### Bacterial strains and culture conditions

*Escherichia coli* XL1-Blue was used for gene cloning and plasmid maintenance. *L. citreum* CB2567 strain was used for library screening and production of recombinant proteins. *E. coli* was grown in Luria–Bertani (LB) broth (BD, Franklin Lakes, NJ, USA) at 37 °C with shaking (200 rpm). *L. citreum* was cultured in Lactobacilli MRS broth (BD) at 30 °C with shaking (200 rpm). The appropriate antibiotics were added to the media: 100 mg/L ampicillin (Ap) for *E. coli* and 10 mg/L of chloramphenicol (Cm) for *L. citreum*.

### Plasmid manipulation

The plasmids and primers used in this work are listed in Tables 1 and 2. Polymerase chain reaction (PCR) was done with the C1000™ Thermal Cycler (Bio-Rad, Richmond, CA, USA) and PrimeSTAR HS polymerase (Takara Bio Inc., Shiga, Japan). pCB4170, which is an *E. coli*-*Leuconostoc* shuttle vector, containing the P710 promoter isolated from *Leuconostoc mesenteroides* ATCC 8293 chromosomal DNA (NCBI accession number NC_008531.1) was used as a backbone plasmid for gene expression [14]. For the expression of the sfGFP, the gene was amplified from PRM GFP (Addgene plasmid 40127) by PCR with the primers 710 sfGFP-F, and sfGFP-R, and after digestion with *Hin*dIII and *Pst*I, the PCR fragment was ligated into pCB4170 to yield pCB4170-sfGFP (Fig. 1a). The pCB4270 plasmid was constructed from pCB4270-sfGFP which was isolated by library screening. Both pCB4270-sfGFP and pCB4170 were digested with *Eco*RI and *Bam*HI, and the short fragment (1.6 Kb) of pCB4270-sfGFP and the long fragment (4 Kb) of pCB4170 were ligated to yield pCB4270. To produce the glutathione-S-transferase (GST) in *L.* citreum, the GST gene was amplified from pJH11 [31] by PCR with the primers gst-F and gst-R, and after digestion with *Sal*I and *Pst*I, the PCR product was cloned into pCB4170 and pCB4270 yielding pCB4170-GST and pCB4270-GST, respectively. For secretory production of the α-amylase, the α-amylase gene with its own signal peptide gene was amplified from *Lactobacillus amylovorus* KCTC 3597 by PCR with the primers amy-F and amy-R. The PCR product was digested with *Sal*I and *Pst*I restriction enzymes and then cloned into pCB4170 and pCB4270 yielding pCB4170-Amy and pCB4270-Amy, respectively. All cloning work was done in *E. coli* XL1-Blue, and the constructed plasmids were transformed to *L. citreum* CB2567 by electroporation (25 μF, 1.0 kV, and 400 Ω) [32]. All DNA manipulations, including restriction enzyme digestion, ligation and agarose gel electrophoresis were performed according to standard procedures [33].

**Table 1:** List of bacterial strains, plasmids used in this study

Strain or plasmids	Relevant characteristics	References
Strains		
*E. coli* XL1-Blue	*recA1 endA1 gyrA96 thi*-*1 hsdR17 supE44 relA1 lac [F´ proAB lacI* ^*q*^ *ZDM15 Tn10 (Tet* ^*r*^ *)]*	Stratagene^a^
*L. citreum* CB2567	Wild type	[39]
*L. amylovorus*	Wild type	KCTC 3597
Plasmids		
pJH11	GST-fused PA domain 4, Amp^r^	[31]
pCB4170	*E. coli*-*L. citreum* shuttle vector	[14]
pCB4270	pCB4170 mutant	This study
pCB4170-sfGFP	pCB4170 derivative, P_710_, sfGFP	This study
pCB4170-GST	pCB4170 derivative, P_710_, GST	This study
pCB4170-Amy	pCB4170 derivative, P_710_, α-amylase	This study
pCB4270-sfGFP	pCB4270 derivative, P_710_, sfGFP	This study
pCB4270-GST	pCB4270 derivative, P_710_, GST	This study
pCB4270-Amy	pCB4270 derivative, P_710_, α-amylase	This study

^a^Stratagene (La Jolla, CA, USA)

**Table 2:** List of primers used in this study

Primers	Sequence (5′ to 3′)
710 sfGFP-F	CTGTTATAATTGATATAACCAGAAGTCGACTAGCTTGAA AGGATAGAAAAAATGAGCAAAGGAGAAGAACTTTTCAC
sfGFP-R	AAGGTTCTGCAGGCGGCCGCTTATTATTTGTAGAGCTCATCCATGC
32-F	TTCCAAGGATCCCCGGGTACCG
32-R	AAGGCCGAATTCGAGCTCGGTACCATG
gst-F	AACCTTGTCGACGAAAGGATAGAAAAAATGTCCCCTATACTAGGTTATTG
gst-R	AAGGTTCTGCAGTTATTAATCCGATTTTGGAGGATGGTC
amy-F	AACCTTGTCGACTTTCAATATTTTAATAAAGGGGGCAGTAAAAAGTG
amy-R	AAGGTTACTAGTGCTGGTATCGGCTTACG
pho-F	ACACAACTAACCGTCAATGGATG
pho-R	CCTTCAAGCCAACCTTCAGC
cm-F	TTGAAGTCATTCTTTACAGGAGTC
cm-R	CGGAGAGTTAGGTTATTGGGATA
C4690T-F	AGGTCACCAAGTCCTAACCTTTGCAGGGGTTGAGGTC
C4690T-R	GACCTCAACCCCTGCAAAGGTTAGGACTTGGTGACCT

### Construction of the random library

In the plasmid pCB4170-sfGFP, the 32-region (1587 bp) which is responsible for the replication in *L. citreum,* was randomized by error-prone PCR with two primers 32-F and 32-R. For each PCR reaction, 4 μL of forward and reverse primers (20 μM), 10.9 μL of dNTP (3.5 μL of dATP (10 μM), 4.0 μL of dCTP (10 mM), 2.0 μL of dGTP (10 mM) and 1.4 μL of dTTP (100 mM)), 5 μL of BSA (0.1 g/L), 10 μL of MgCL_2_ free 10X PCR buffer (Takara Bio. Inc., Shiga, Japan), 10 μL of MgCl_2_ (25 μM), 2 μL of MnCl_2_ (5 mM), 53.1 μL of distilled water and 1 μL of template DNA (pCB4170) and 0.024 U of Taq DNA polymerase (Takara Bio. Inc.) were mixed [34, 35]. PCR was done as follows: 30 cycles of 96 °C for 1 min., 58 °C for 30 s and 72 °C for 1.5 min. PCR product was digested with *Eco*RI and *Bam*HI restriction enzymes and cloned into the same restriction enzyme sites of pCB4170-sfGFP. Ligation was done at 16 °C for 12 h. The ligated plasmids were transformed to the *E. coli* XL1-Blue competent cell by electroporation (Bio-Rad). Transformed cells were recovered on LB agar plates with 100 mg/L ampicillin at 37 °C for 12 h. The library plasmids were purified with GeneAll^®^ Hybrid-Q™ Plasmid Rapidprep kit (GeneAll, Seoul, Korea) and transformed into *L. citreum* CB2567 by electroporation. Transformed cells were recovered on MRS agar plates with 10 mg/L chloramphenicol at 30 °C for 48 h.

### FACS screening

The random library of plasmids was analyzed and sorted by fluorescent activated cell sorter (MoFlo XDP, Beckman Coulter, Inc., Miami, FL, USA) with a 488 nm argon laser. The library was cultured overnight at 30 °C in MRS media containing chloramphenicol (10 mg/L). Cells were transferred to fresh MRS media at a 1:50 dilution and cultivated for 8 h at 30 °C with shaking. Cells were harvested by centrifugation (3300*g*, 10 min, 4 °C) and washed twice with phosphate-buffered saline (PBS, 135 mM NaCl, 2.7 mM KCl, 4.3 mM Na_2_HPO_4,_ pH 7.2) and resuspended in the same buffer. Suspended cells were screened by the FACS sorter based on high fluorescence intensity detection through a 530/40 band-pass filter for the GFP emission spectrum. Sorted cells were cultivated on MRS agar plates with chloramphenicol (10 mg/L) at 30 °C for 24 h. All grown colonies were collected and transferred to MRS liquid media for next round sorting.

### Plasmid segregational stability test

The plasmid segregational stability of pCB4170 and pCB4270 was investigated under non-selective growth condition. At 12 h intervals, the cultured cells were transferred to antibiotic-free MRS media with a 1/50 dilution factor during the exponential growth phase. Culture samples were diluted and plated onto MRS plates with and without chloramphenicol to determine colony forming unites (CFU) at every 24 h. The plasmid stability was determined until 80 generations using duplicates.

### Determination of copy number by quantitative PCR

The relative plasmid copy number (PCN) was calculated with the quantitative PCR method. Amplification and analysis were done with a LightCycler instrument (Roche, Basel, Switzerland) and one step SYBR PrimeScript kit (TAKARA BIO, Inc.). The single copy gene of phosphoketolase (*pho*) from *L. citreum* chromosomal DNA was used as the control gene. The chloramphenicol gene (Cm) gene was used for the detection of the plasmid. After cultivation, the same amount of cells was harvested by centrifugation, and whole genomic DNA was extracted from the cells with the MasterPure™ DNA6 Purification Kit (Epicentre^®^ an illumine company, Madison, WI, USA) according to the manufacturer’s instructions. The value of the quantification cycle (C_q_) of the target genes was determined by the LightCycler^®^ 96 Software and averaged with triplicates. The amplification efficiency of each gene was measured with a tenfold dilution of the pCB4170-sfGFP plasmid (0.1–100 ng/μl). PCN was determined with Cq value and the amplification efficiency of the plasmid (−p) and chromosomal gene (−c). The amplification efficiency (E) was calculated from the slope of the standard curve (${\text{E}} = 10^{{\left( { - 1/{\text{slope}}} \right)}}$). Using the calculated amplification efficiency value, the plasmid copy number was determined with the following equation: PCN = (E_c_)^Cqc^/(E_p_)^Cqp^ [36].

### Protein preparation and analysis

After flask cultivation for 12 h, cells were harvested by centrifugation at 3300*g* for 10 min. at 4 °C. The harvested cells were washed with PBS and suspended in the same buffer. After cell disruption by sonication (20 min with 5-s pulses and 5-s intervals, 20 % amplitude), soluble lysate was prepared by centrifugation (9300*g* for 10 min. at 4 °C). Protein samples were analyzed by 12 % sodium dodecyl sulfate–polyacrylamide gel electrophoresis (SDS-PAGE) and western blotting. In the SDS-PAGE analysis, cell lysates were mixed with SDS-PAGE sample buffer and boiled for 5 min at 100 °C. After the electrophoresis, the gels were stained with Coomassie brilliant blue dye (50 % (*v/v*) methanol, 10 % (*v/v*) acetic acid, 1 g/L Coomassie brilliant blue R-250) for 1 h and de-stained with a destaining solution (10 % (*v/v*) acetic acid, 10 % (*v/v*) methanol). The densitometric analysis of protein bands was performed by ImageJ software (http://rsb.info.nih.gov/ij). For the western blotting, all the proteins on the gel were transferred to a polyvinyl difluoride (PVDF) membrane (Roche) after gel electrophoresis. The membrane was blocked with 5 % (*w/v*) skim-milk in Tris-buffered saline with Tween-20 solution (TBS-T; 24.7 mM Tris, 137 mM NaCl, 2.7 mM KCl and 0.05 % Tween-20) for 1 h at room temperature. The membrane was incubated with horseradish peroxidase (HRP)-conjugated goat ANTI-GFP antibody (Abcam, Cambridge, UK) or HRP-conjugated ANTI-GST antibody (GE Healthcare, Uppsala, Sweden) that were 1:5000 diluted with TBS-T buffer containing 5 % skim-milk for 1 h. Each membrane was washed 5 times with TBS-T. For immunodetection of the target protein, enhanced chemiluminescence reagent (ECL Prime, GE Healthcare) was used. After the treatment of reagent, the target protein bands were visualized on X-ray films.

### α-amylase activity assay

The activity of α-amylase secreted into the extracellular media was detected by the reaction between the starch plate and the iodine solution [37]. Overnight cultures of *L. citreum* were spotted onto MRS plates with 0.5 % soluble starch and 10 mg/L chloramphenicol. After incubation at 30 °C for 24 h, 1 mL of Lugol’s iodine was evenly poured onto the plate. Clear halo zones around the colonies indicate starch degradation by secreted α-amylase. The quantitative activity of amylase was detected with the EnzChek^®^ Ultra Amylase Assay Kit (Thermo Fisher Scientific, Waltham, MA, USA) following the manufacturer’s instructions. Culture supernatants from an 8 h cultivation were prepared, and the fluorescence intensity was detected with Infinite^®^ F200 PRO (TECAN, Männedorf, Switzerland) [38]. A standard curve was constructed with α-amylase from *Bacillus Sp*. according to the manufacturer’s instructions.
